# Supplementary material for: Elemental Composition and Degradation Rate Impact the Biocompatibility of Copper Chalcogenide Nanocrystals
Source: bioRxiv. 2026 Feb 8:2025.12.17.695045. Originally published 2025 Dec 20. Preprint. [Version 2] doi: 10.64898/2025.12.17.695045 (PMC12724650; doi:10.64898/2025.12.17.695045)
Supplement: Supplement 1 [file media-1.pdf]

Supporting Information

Elemental Composition and Degradation Rate Impact the Biocompatibility of Copper Chalcogenide Nanocrystals

Xingjian Zhong[1, 2], G. Perry Katsarakes[1], Savani Nagarkar[2], Allison M. Dennis[2, 3*]

1. Department of Biomedical Engineering, Boston University, Boston 02215
2. Department of Chemical Engineering, Northeastern University, Boston 02115
3. Department of Bioengineering, Northeastern University, Boston 02115

*Corresponding Author: Allison M. Dennis, a.dennis@northeastern.edu

Figure S1. Size distribution of copper chalcogenide nanocrystals

Figure S2. Absorbance spectrum of micelle-encapsulated $Cu_{2-x}S$

Figure S3. Dynamic light scattering (DLS) hydrodynamic diameter and ζ-potential measurements

Figure S4. Cation release comparison between compositions

Figure S5. Control experiment results of apoptosis/necrosis assay measured with flow cytometry

Figure S6. Summary of apoptosis results

Table S1. Ionic composition of simulated body fluid (SBF) and artificial lysosomal fluid (ALF)

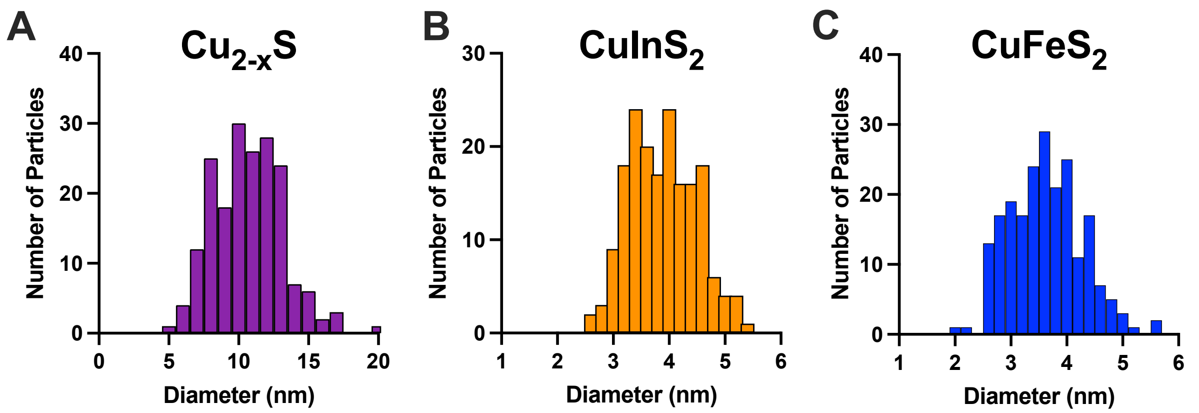

Figure S1. Size distribution of copper chalcogenide nanocrystals measured from TEM images. Histograms show particle size distributions for (**A**) $Cu_{2-x}S$, (**B**) $CuInS_2$, and (**C**) $CuFeS_2$. Mean ± standard deviation of particle diameters: $Cu_{2-x}S$ = 10.7 ± 2.5 nm (n = 187); $CuInS_2$ = 3.9 ± 0.6 nm (n = 182); and $CuFeS_2$ = 3.6 ± 0.7 nm (n = 213).

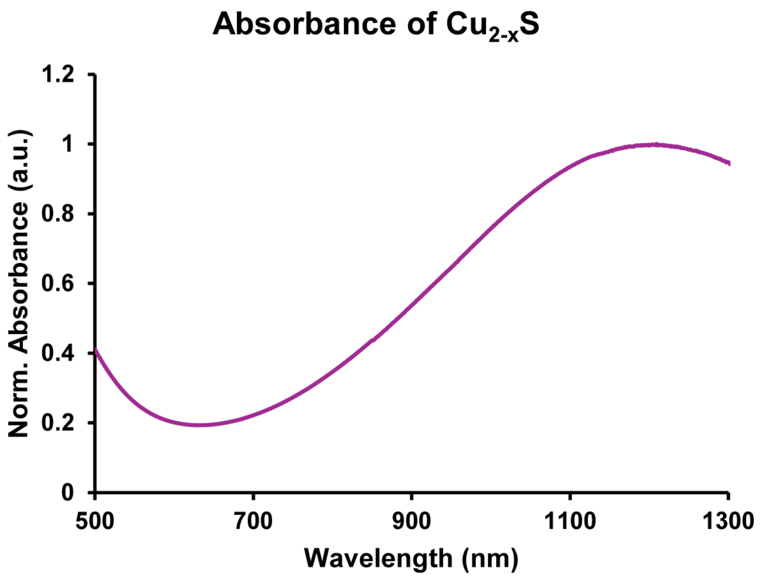

Figure S2. Absorbance spectrum of micelle-encapsulated $Cu_{2-x}S$ nanocrystals into the near-infrared range. Data is normalized to absorbance peak. Absorbance at wavelengths > 1300 nm is not shown due to large artifacts from water absorbance.

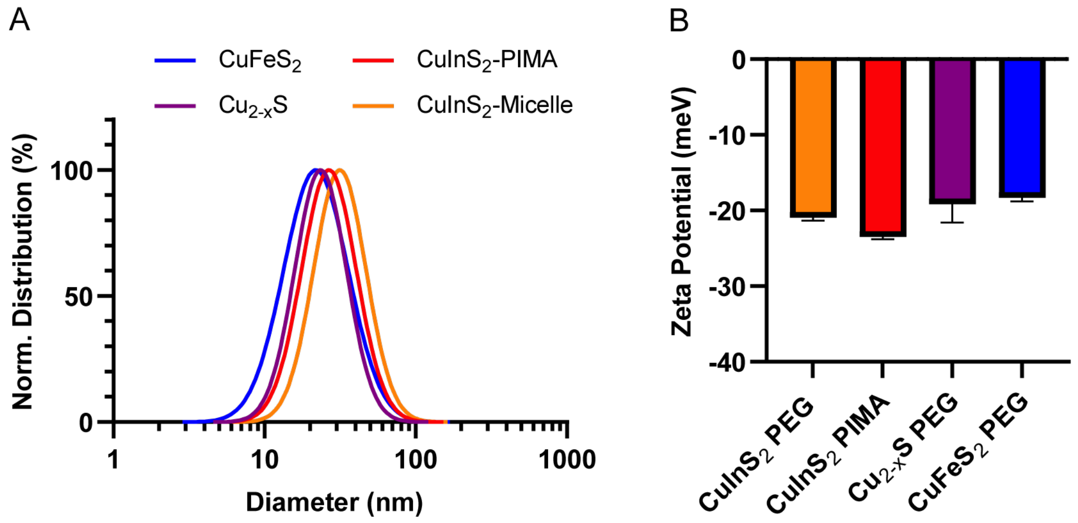

Figure S3. Dynamic light scattering (DLS) hydrodynamic diameter and ζ-potential measurements. (**A**) Number-weighted DLS measurements on log-normal plot. Average hydrodynamic diameter based on number-weighted peaks: $CuFeS_2$ (22.8± 1.8 nm); $Cu_{2-x}S$ (26.0 ± 4.9 nm); $CuInS_2$-Micelle (36.0 ± 4.4 nm); $CuInS_2$-PIMA (30.8 ± 1.2 nm). (**B**) ζ-potential measurements of particles. DLS and Zeta were measured with NanoBrook 90Plus PALS (Brookhaven, NH) with n = 3 repeats.

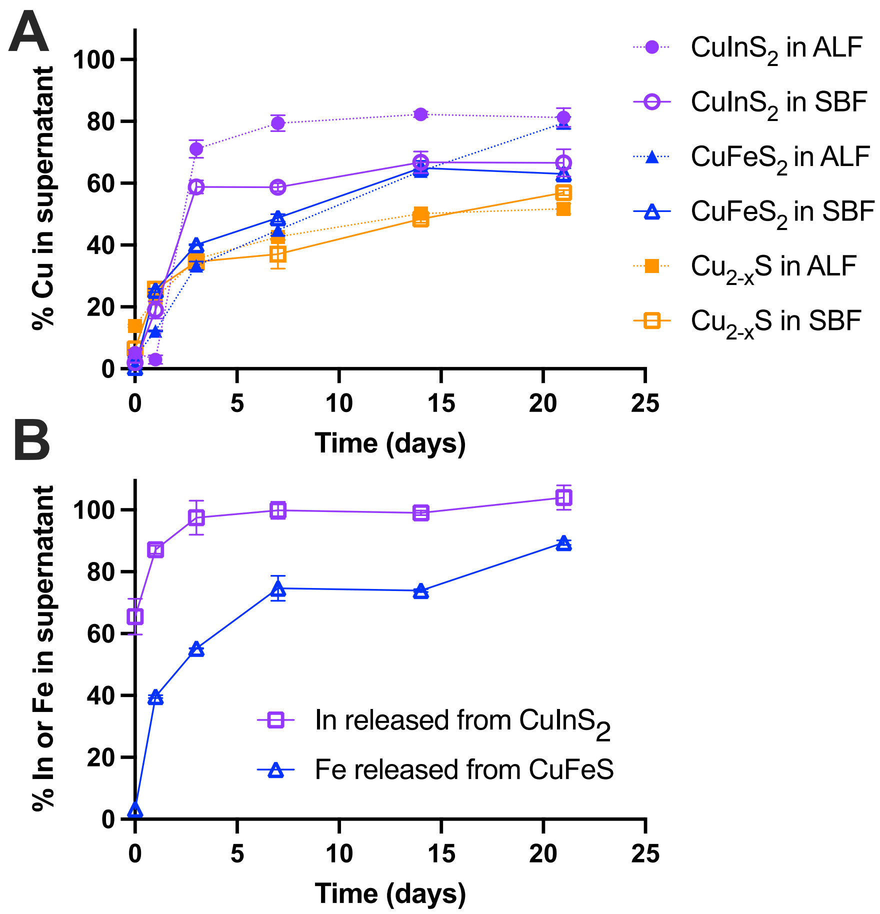

Figure S4. Cation release comparison between compositions. (**A**) Release of copper into the supernatant upon incubation of micelle-encapsulated copper chalcogenides in SBF and ALF. (**B**) Release of indium and iron into the supernatant upon incubation of micelle-encapsulated NCs in SBF.

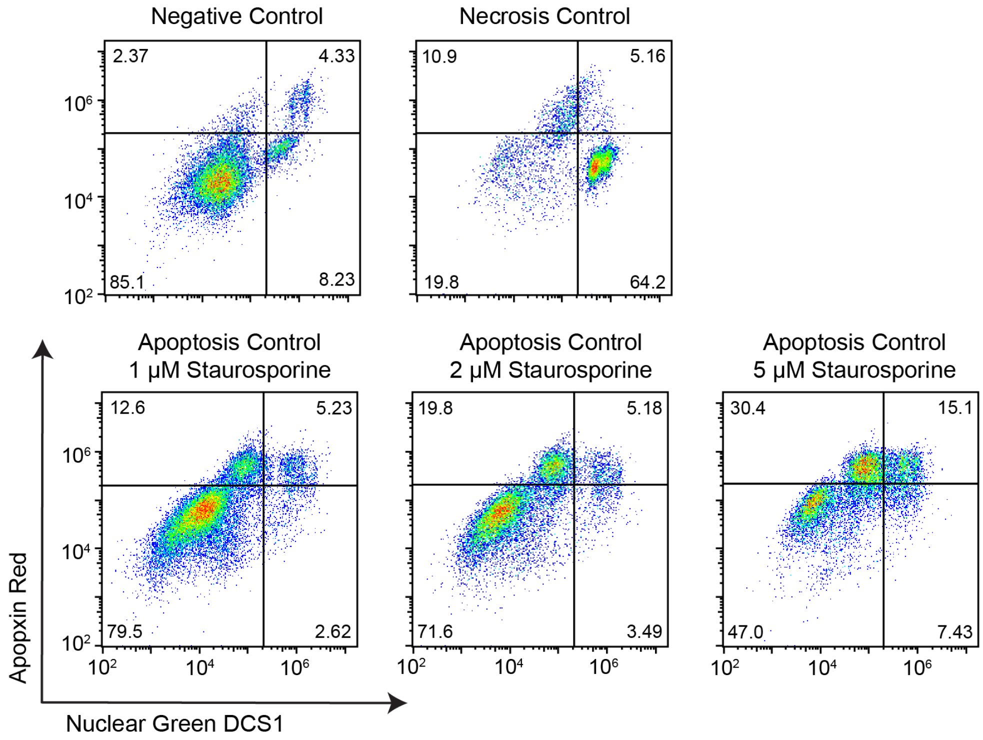

Figure S5. Control experiment results of apoptosis/necrosis assay measured with flow cytometry. Particles were incubated with pH 7.4 PBS buffer for negative control; 90% ethanol for 60 seconds for necrosis positive control; 1 – 5 μM staurosporine for 24 hr for apoptosis positive control.

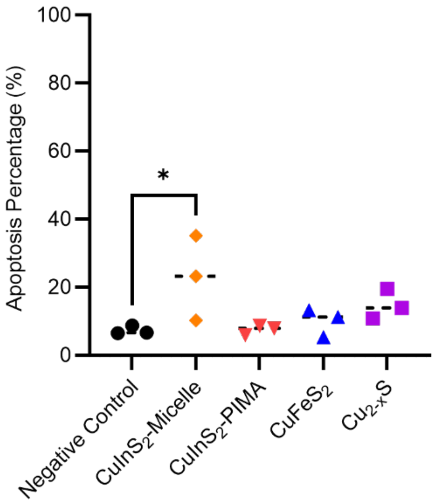

Figure S6. Summary of apoptosis results. Comparison between different experimental groups and negative control. *P < 0.05.

Table S1. Ionic composition of simulated body fluid (SBF)[1,2] and artificial lysosomal fluid (ALF),[3] adapted from previous reports**.**

Ions	***Simulated Body Fluid (SBF) (mM)***	***Artificial Lysosomal Fluid (ALF) (mM)***
Na^+	138	208.4
Ca^{2+}	2.6	-
Mg^{2+}	1.5	0.5
K^+	5	-
Cl^-	148.8	55.8
SO_4^{2-}	0.5	0.3
HPO_4^{2-}	1	-
$H_2PO_4^-$	-	0.7
Tris	50	-
Hydrochloric acid	40	-
Tartrate	-	0.4
Lactate	-	0.8
Pyruvate	-	0.8
Citric Acid	-	108
pH	7.4	4.5

References

(1) Marques, M. R. C.; Loebenberg, R.; Almukainzi, M. Simulated Biological Fluids with Possible Application in Dissolution Testing. *Dissolution Technol.* **2011**, *18* (3), 15–28. https://doi.org/10.14227/DT180311P15.

(2) Kays, J. C.; Saeboe, A. M.; Toufanian, R.; Kurant, D. E.; Dennis, A. M. Shell-Free Copper Indium Sulfide Quantum Dots Induce Toxicity *in Vitro* and *in Vivo*. *Nano Lett.* **2020**, *20* (3), 1980–1991. https://doi.org/10.1021/acs.nanolett.9b05259.

(3) Stopford, W.; Turner, J.; Cappellini, D.; Brock, T. Bioaccessibility Testing of Cobalt Compounds. *J. Environ. Monit.* **2003**, *5* (4), 675–680. https://doi.org/10.1039/B302257A.
