# Supplementary material for: Elemental Composition and Degradation Rate Impact the Biocompatibility of Copper Chalcogenide Nanocrystals
Source: bioRxiv. 2025 Dec 20:2025.12.17.695045. Preprint. [Version 1] doi: 10.64898/2025.12.17.695045 (PMC12724650; doi:10.64898/2025.12.17.695045)
Supplement: Supplementary file 1 [file media-1.pdf]

Elemental Composition and Degradation Rate Impact the Biocompatibility of Copper Chalcogenide Nanocrystals

Xingjian Zhong[1, 2], G. Perry Katsarakes[1], Savani Nagarkar[2], Allison M. Dennis[2, 3*]

1. Department of Biomedical Engineering, Boston University, Boston 02215
2. Department of Chemical Engineering, Northeastern University, Boston 02115
3. Department of Bioengineering, Northeastern University, Boston 02115

*Corresponding Author: Allison M. Dennis, a.dennis@northeastern.edu

Supplementary Information

Figure S1: Absorbance spectrum of micelle-encapsulated $Cu_{2-x}S$ into the near-infrared range.

Figure S2: Dynamic light scattering and Zeta potential measurements.

Figure S3: Degradation comparison between $CuInS_2$ with different surface coatings.

Figure S4: Control experiment results of apoptosis/necrosis assay measured with flow cytometry.

Figure S5: Comparison between apoptosis population of different experimental groups and negative control.

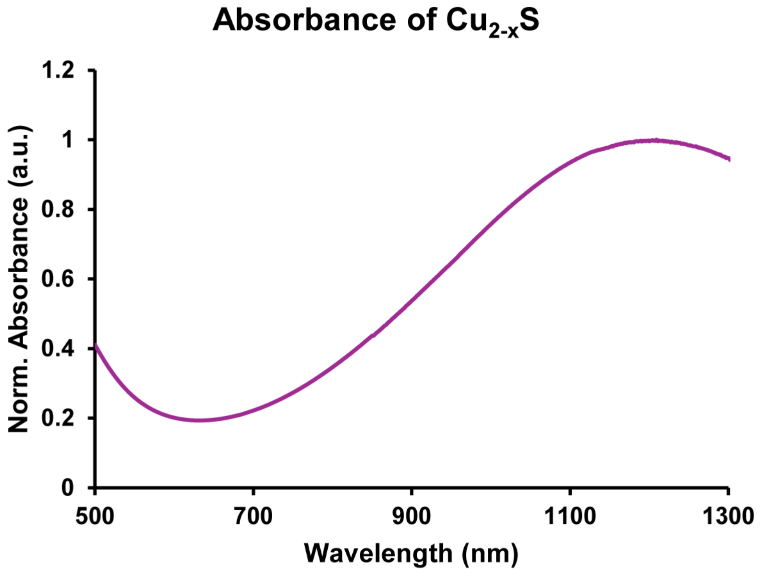

Figure S1. Absorbance spectrum of micelle-encapsulated $Cu_{2\text{-}x}S$ nanocrystals into the near-infrared range. Data is normalized to absorbance peak. Absorbance at wavelengths > 1300 nm is not shown due to large artifacts from water absorbance.

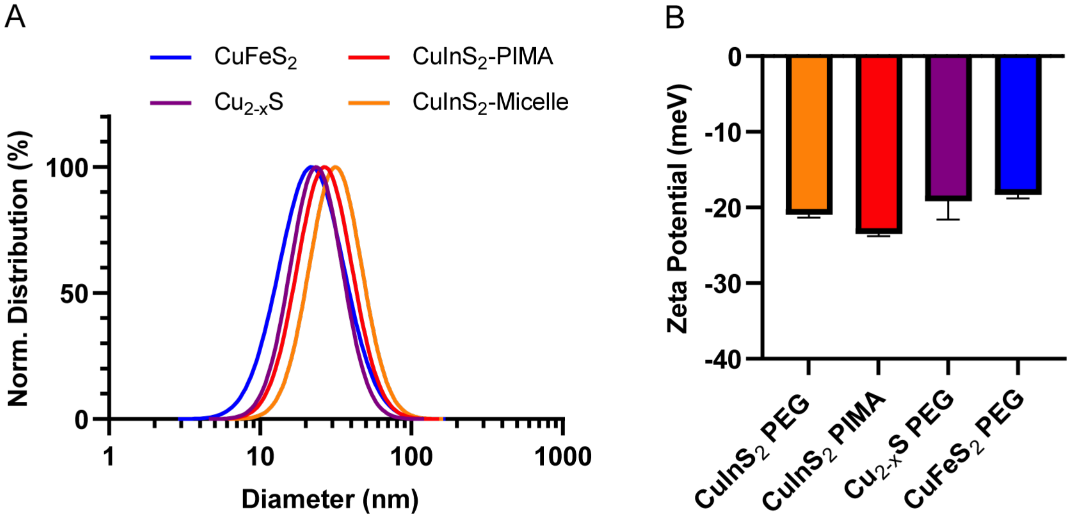

Figure S2. Dynamic light scattering (DLS) hydrodynamic diameter and ζ-potential measurements. (**A**) Number-weighted DLS measurements on log-normal plot. Average hydrodynamic diameter based on number-weighted peaks: $CuFeS_2$ (22.8± 1.8 nm); $Cu_{2\text{-}x}S$ (26.0 ± 4.9 nm); $CuInS_2$-Micelle (36.0 ± 4.4 nm); $CuInS_2$-PIMA (30.8 ± 1.2 nm). (**B**) ζ-potential measurements of particles. DLS and Zeta were measured with NanoBrook 90Plus PALS (Brookhaven, NH) with n = 3 repeats.

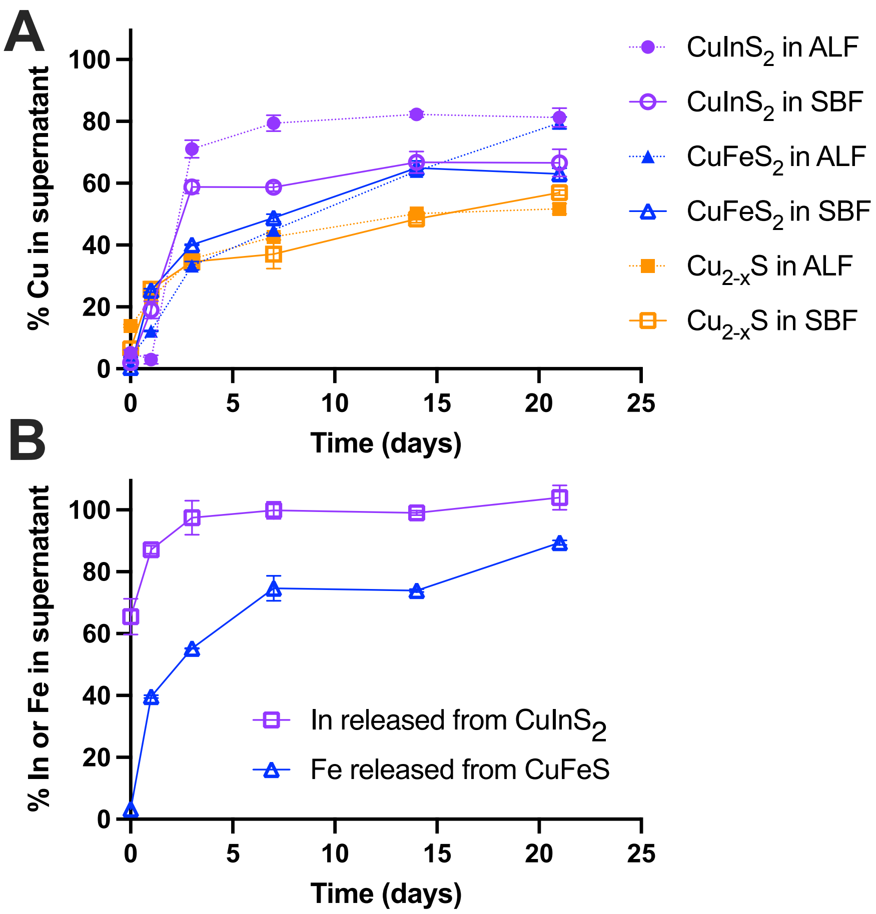

Figure S3. Cation release comparison between compositions. (**A**) Release of copper into the supernatant upon incubation of micelle-encapsulated copper chalcogenides in SBF and ALF. (**B**) Release of indium and iron into the supernatant upon incubation of micelle-encapsulated NCs in SBF.

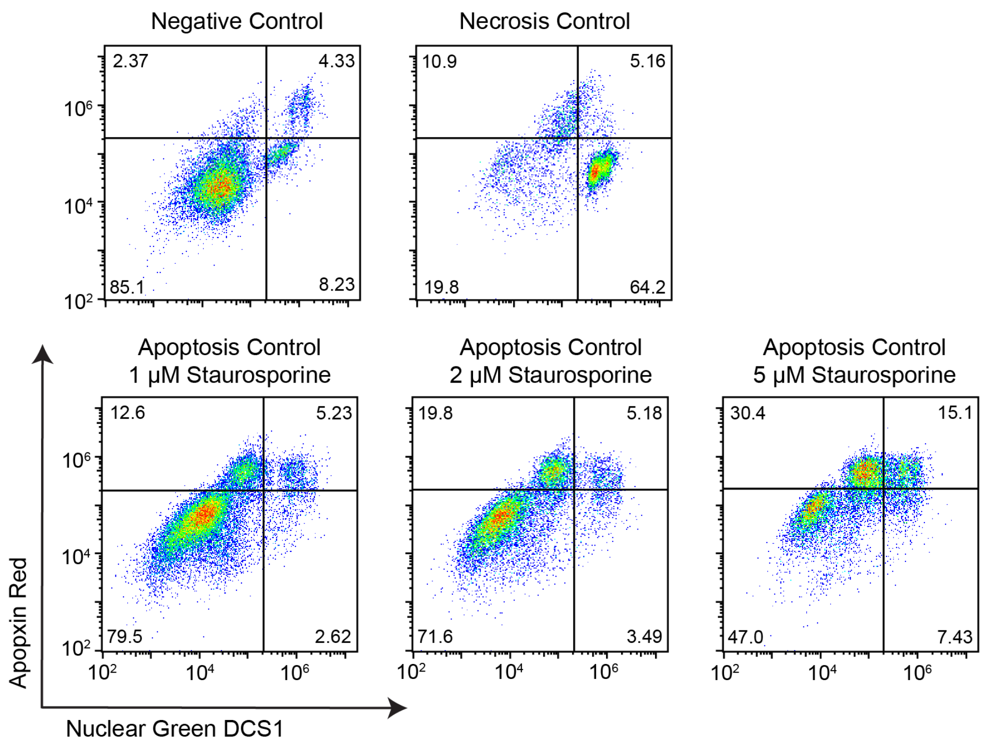

Figure S4. Control experiment results of apoptosis/necrosis assay measured with flow cytometry. Particles were incubated with pH 7.4 PBS buffer for negative control; 90% ethanol for 60 seconds for necrosis positive control; 1 – 5 μM staurosporine for 24 hr for apoptosis positive control.

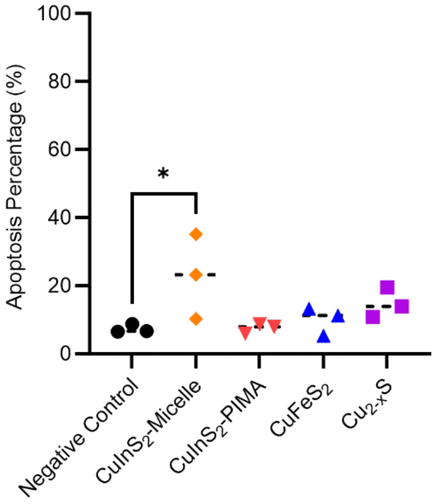

Figure S5. Comparison between apoptosis population of different experimental groups and negative control. *P < 0.05.
